# Training a deep learning model for single-cell segmentation without manual annotation

**DOI:** 10.1038/s41598-021-03299-4

**Published:** 2021-12-14

**Authors:** Nizam Ud Din, Ji Yu

**Affiliations:** Center for Cell Analysis and Modeling, UConn Health, 400 Farmington, Farmington, CT 06030 USA

**Keywords:** Image processing, Machine learning

## Abstract

Advances in the artificial neural network have made machine learning techniques increasingly more important in image analysis tasks. Recently, convolutional neural networks (CNN) have been applied to the problem of cell segmentation from microscopy images. However, previous methods used a supervised training paradigm in order to create an accurate segmentation model. This strategy requires a large amount of manually labeled cellular images, in which accurate segmentations at pixel level were produced by human operators. Generating training data is expensive and a major hindrance in the wider adoption of machine learning based methods for cell segmentation. Here we present an alternative strategy that trains CNNs without any human-labeled data. We show that our method is able to produce accurate segmentation models, and is applicable to both fluorescence and bright-field images, and requires little to no prior knowledge of the signal characteristics.

## Introduction

Automated cellular segmentation from optical microscopy images is a critical task in many biological researches that rely on single-cell analysis. Traditional approaches to cell segmentation rely on manually-crafted feature definitions that allow the algorithmic recognition of cellular area and cell border^1,2^. Unfortunately manual feature definitions are usually highly context-specific and require task-dependent tuning to work well. For example, an algorithm that is well-optimized for a specific membrane fluorescent marker will not work on images of a different fluorescent marker, nor on bright field images. Switching to a different cell type (with different morphological features), changing imaging modality (e.g., from epi-fluorescence to confocal), or even imaging settings (e.g. changing the objective) often requires a redesign and/or reoptimization of the segmentation algorithm. Therefore, there is a need for more generic, “turn-key” solutions for this task.

Convolutional neural networks (CNN) have been shown to be highly efficient on various kinds of image processing tasks, including semantic and instance segmentations^3–6^. In particular, various networks with an encoder-decoder architecture (e.g., UNet) have achieved remarkable pixel-level accuracy in the segmentation of objects, including biological cells^7–11^. However, to train a CNN for the segmentation task, one typically needs a significant amount of manually labeled training images, in which cell areas and/or cell boundaries are marked by human operators. However, high quality annotations of this type are scarce in general. More recently, methods for unsupervised semantic segmentation started to emerge^12–17^. These methods try to find a discriminative pixel embedding that can be linked to the segmentation classes. Since no external class labels are available, the models learn the representation by performing some pretext tasks, such as to maintain spatial coherence^12,13^ or image contour^16^. In some cases, additional intra-class samples were generated by geometric transformations^12^ or from a separate model^16^. Additionally, metric learning approaches were employed to minimize intra-class variations and maximize inter-class variation. This is achieved by using pairwise losses, e.g. noise contrastive loss^13,16^, by maximizing mutual information^12^, or by applying a mixture model^15^. Despite these advances, so far none of the techniques proposed has been applied to the task of cell segmentation yet. One problem stems from the mismatch in the design goal. Most of the proposed methods focus on general machine learning tasks covering a large range of visual domains, while microscopy images of cells have relatively simple visual domains, e.g., cell versus background. Conversely, the proposed methods generally do not account for separating touching instances of the same class, which makes them not applicable to the majority of the biological tissues, where cells are closely clumped together. One workaround is to classify pixels as either cell-boundary, cell-interior or background, and rely on post-processing to obtain segmentations for individual cells. However, this approach often exhibits an under-segmentation bias^8^. As a result, CNN-based approaches remain uncompetitive versus traditional segmentation approaches in real-life biological applications.

Here we propose a CNN segmentation method that is specifically suitable for situations where cells are tightly clumped together. In addition, our approaches differ from previous attempts in that we use a maker-controlled segmentation algorithm, taking a note from traditional cell segmentation pipelines. Many conventional segmentation algorithms (e.g., watershed^18^) are known to have an over-segmentation bias when the images are contaminated with noise. One way to correct for the bias is to provide a set of specific marker positions, depicting the approximate locations of each cell. Indeed, it is a common practice to use two-channel imaging data for cell segmentation, one channel for imaging nucleus and the other for the whole cell. The nucleus images are useful for computing a rough cell position, and the whole cell images provide information for determining cell boundaries. This approach is used in some of the most widely-used cell segmentation softwares, such as CellProfiler^19^ and Ilastik^20^. The success of this approach derives partly from the fact that nucleus images have simple morphological features and therefore are relatively easy to analyze. Nucleus identification and/or segmentation is a heavily researched topic; multiple algorithms exist in the literature with good performances^21–25^. The main challenge in cell segmentation is to devise reliable feature representations that can recognize cell boundaries with a high accuracy.

**Figure 1:**
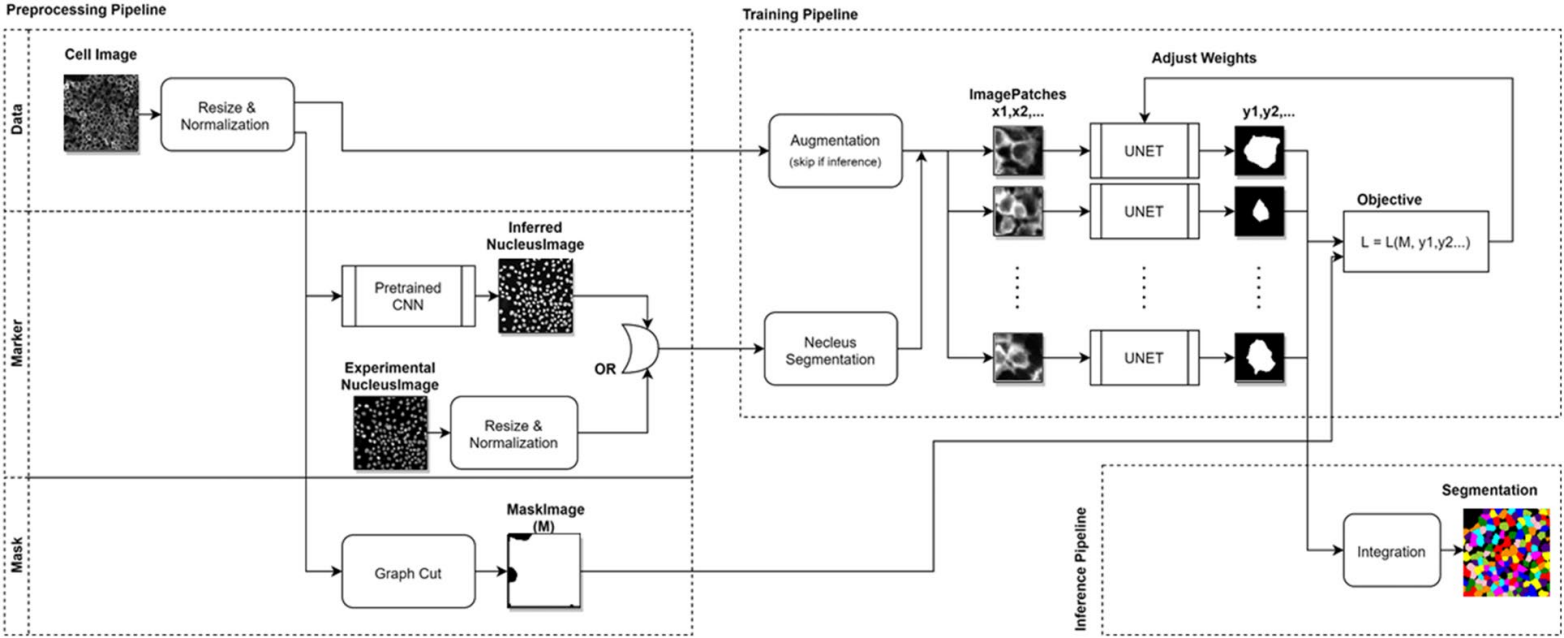
Schematic outline of the CNN segmentation algorithm. The overall process comprises three components: the preprocessing pipeline, which computes the marker location and image mask from the inputs, the training pipeline which extract image patches to train a CNN model for segmentation, and the integration pipeline that takes the single patch output from the CNN and create whole-field segmentation results. This figure is produced using the software diagrams.net (v14.6.11).

We propose to adopt the marker-controlled segmentation approach, but applying it to the CNN-based segmentation methodology. We will first generate marker locations from nucleus images, but use a CNN to capture semantic features of whole cells. Specifically, we designed our neural network to perform segmentation on a smaller patch of the input image centered on the marker positions (Fig. 1). This converts the multi-cell segmentation problem to multiple single-cell segmentation problems, which in turn removes the under-segmentation bias as long as the nuclei markers were correctly computed. In addition, it is important to note that the “nucleus image” does not have to be one that is experimentally acquired. Instead, we will also demonstrate an alternative approach, in which we generate synthetic “nucleus images” from the normal whole cell images, by using a pretrained CNN model. This technique is similar to the method first demonstrated by Ounkomol et.al^26^, in which they showed that CNNs can be trained to map one image modality (e.g. bright-field image) to a different one (e.g., fluorescence images of plasma membrane, nucleus etc.).

A more important consequence of the marker-controlled segmentation approach is that it allows us to devise a method to train segmentation CNNs (Fig. 1) in an unsupervised manner, thus removing the need to manually label the training dataset. The key for achieving this is to design a custom objective function:

1 $$L\left(M,{y}^{1},{y}^{2},\dots ,{y}^{n}\right)=-\sum_{{d}_{0},{d}_{1}}\sum_{i}{y}_{{d}_{0},{d}_{1}}^{i}-\lambda \sum_{{d}_{0},{d}_{1}}\sum_{i\ne j}{y}_{{d}_{0},{d}_{1}}^{i}\mathrm{log}{y}_{{d}_{0},{d}_{1}}^{j}-\beta \sum_{{d}_{0},{d}_{1}}\sum_{i}{y}_{{d}_{0},{d}_{1}}^{i}(1-{M}_{{d}_{0},{d}_{1}})$$

Here, ${y}^{i}$ represents the CNN output from *i*-th image patch, and ${y}_{{d}_{0},{d}_{1}}^{i}\in (\mathrm{0,1})$ denotes the value of ${y}^{i}$ at a specific pixel location, ${d}_{0,}{d}_{1}$. Interpretation of ${y}^{i}$ is similar to other CNN-based segmentation outputs, i.e., it is the probability of the segmentation mask for the cell. The image patch has a dimension of ${\varvec{k}}\times {\varvec{k}}$, which should be much smaller than the whole image, but bigger than a single cell. Consequently, there will be spatial overlaps between different patches. Our CNN outputs the same spatial dimension as that of its input (Fig. S1). Therefore ${y}^{i}$ should also have a dimension of ${\varvec{k}}\times {\varvec{k}}$. However, to simplify the expression in Eq. (1), we use a notation in which the pixel indexing into ${y}^{i}$, denoted by ${d}_{0,}{d}_{1}$, is allowed over the size of the whole input image. This is possible because we can simply assume ${y}^{i}$ to be 0 at locations outside the specific image patch, which is consistent with probability interpreting of ${y}^{i}$. In other words, ${y}^{i}$ can be viewed as a zero-padded matrix that has the same size as that of the whole input image, but has only non-zero elements within a small ${\varvec{k}}\times {\varvec{k}}$ patch.

The goal of the network training is to minimize the objective function *L*. It is easy to see that the first term is simply to maximize the total area of all segmented pixels from all patches. This is balanced by the second term, which can be viewed as an extension of the cross-entropy loss. In standard cross-entropy loss function, a prediction *p* is checked against a true value *q*, but computing $p\cdot \mathrm{log}q$ as the loss. Here, instead of being compared against a true value, which we do not have, the prediction, ${y}_{{d}_{0}{d}_{1}}^{i}$, is compared against the inverse of the prediction from another patch, $1-{y}_{{d}_{0}{d}_{1}}^{j}$. The end result is that we enforced consistency of segmentations from overlapping patches. By jointly minimizing the first two terms, we entice the CNN to learn semantic features defining the cell boundaries from the image data (Fig. S2). Furthermore, the loss function also uses a third term that gives additional penalties if the segmented pixels exceed the overall cellular area, depicted by the binary mask *M*, therefore preventing the model from including the background pixels in the segmentation. This term provides additional constraints for the model training and helps to improve the overall model accuracy. The binary mask can be generated from the image data using traditional segmentation methods such as Graph Cut. Examples of such masks can be found in Fig. S3. Two hyper parameters, $\lambda$ and $\beta$, control the relative weight of the penalty terms. Finally, the loss function is designed to be fully differentiable; therefore we can use the standard stochastic optimization and back-propagation technique to achieve the training goal.

## Results

We implemented the proposed algorithm using the TensorFlow computational framework and tested its efficacy on both immunofluorescence (anti-phosphortyrosine (pY)) and bright-field microscopy image data. In addition, to compute marker locations, we obtained nucleus images by staining cells with DNA binding dye DAPI. Traditional segmentation algorithms generally do not work on bright-field images and often work poorly on noisy signals such as those in immunofluorescence data. Therefore, our datasets serve as stress tests of the segmentation algorithm by presenting more challenging use cases. To allow quantitative evaluations of the machine segmentation results, we manually segmented 130 cells randomly selected from the three experimental datasets. The manual segmentation is performed by examining all available inputs (immuno-fluorescence, bright-field, and nucleus images) to ensure the highest accuracy.

We train modified UNets (Fig. S1) with the acquired microscopy images to generate a segmentation model. We first used the CellProfiler software to analyze the nucleus image and computed the locations of each detected nucleus as the markers. Using CellProfiler for locating nuclei markers allows us to also use CellProfiler segmentation as a baseline model for whole cell segmentation, and facilitate comparison with our model on an equal footing. The marker locations were used to produce image patches (64 × 64 pixels), each centered around a marker location. The patch size was chosen to be larger than the size of a single cell, thus many patches have significant overlapping areas. The image patches were used as the inputs to train the neural network by minimizing the loss function. The goal is to train the UNet to segment one single cell from each image patch. However, CNN output is dependent on local information only, and is not aware of the absolution positions of the pixel inputs. To ensure the segmentation apply to only one specific cell, we encoded positional information in the input data by including a image of a Gaussian disk (σ = 15) at the marker location as an additional input channel. On top of that, in order to ensure the features learned about cells are symmetric according to image orientation, we randomly flip the patches along two major axes. A corresponding reverse operation was performed on the network output before the computation of the loss function. For each independent dataset input, we optimize a separate segmentation model based on the same underlying network architect and training procedure.

**Figure 2:**
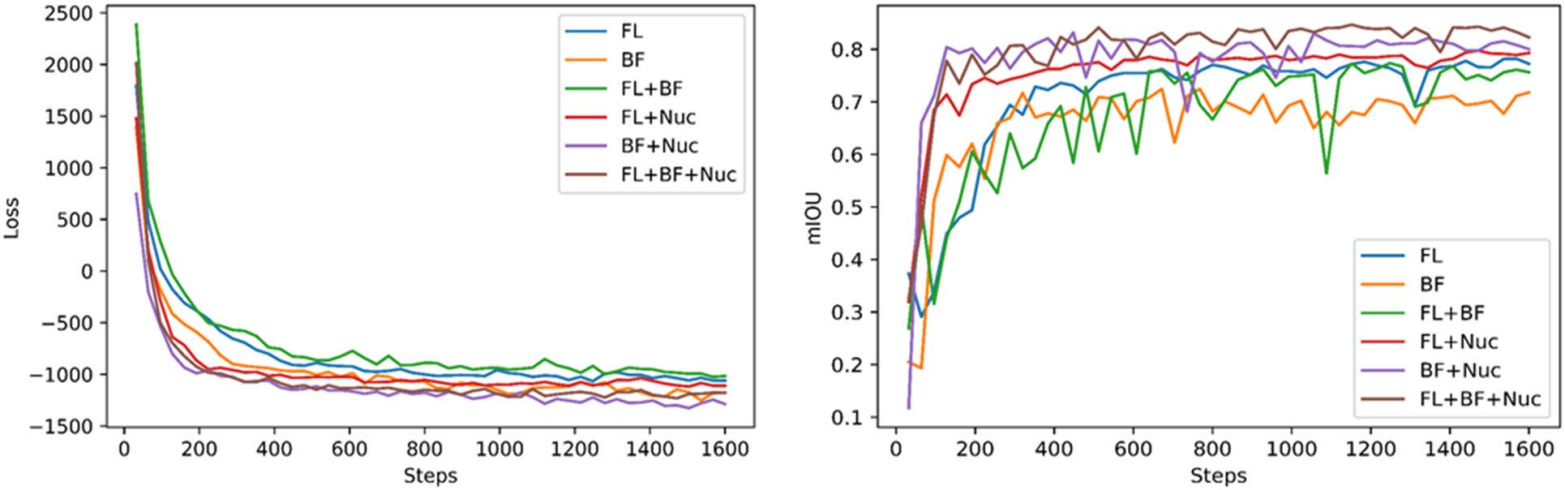
Representative training traces showing the minimization of the loss function (left) and the corresponding optimization of the segmentation accuracies (right) over the self-supervised training process. Multiple models were trained with various combinations of input data, including the immunofluorescence image (FL), the bright-field image (BF) and the DAPI staining image (Nuc).

**Figure 3:**
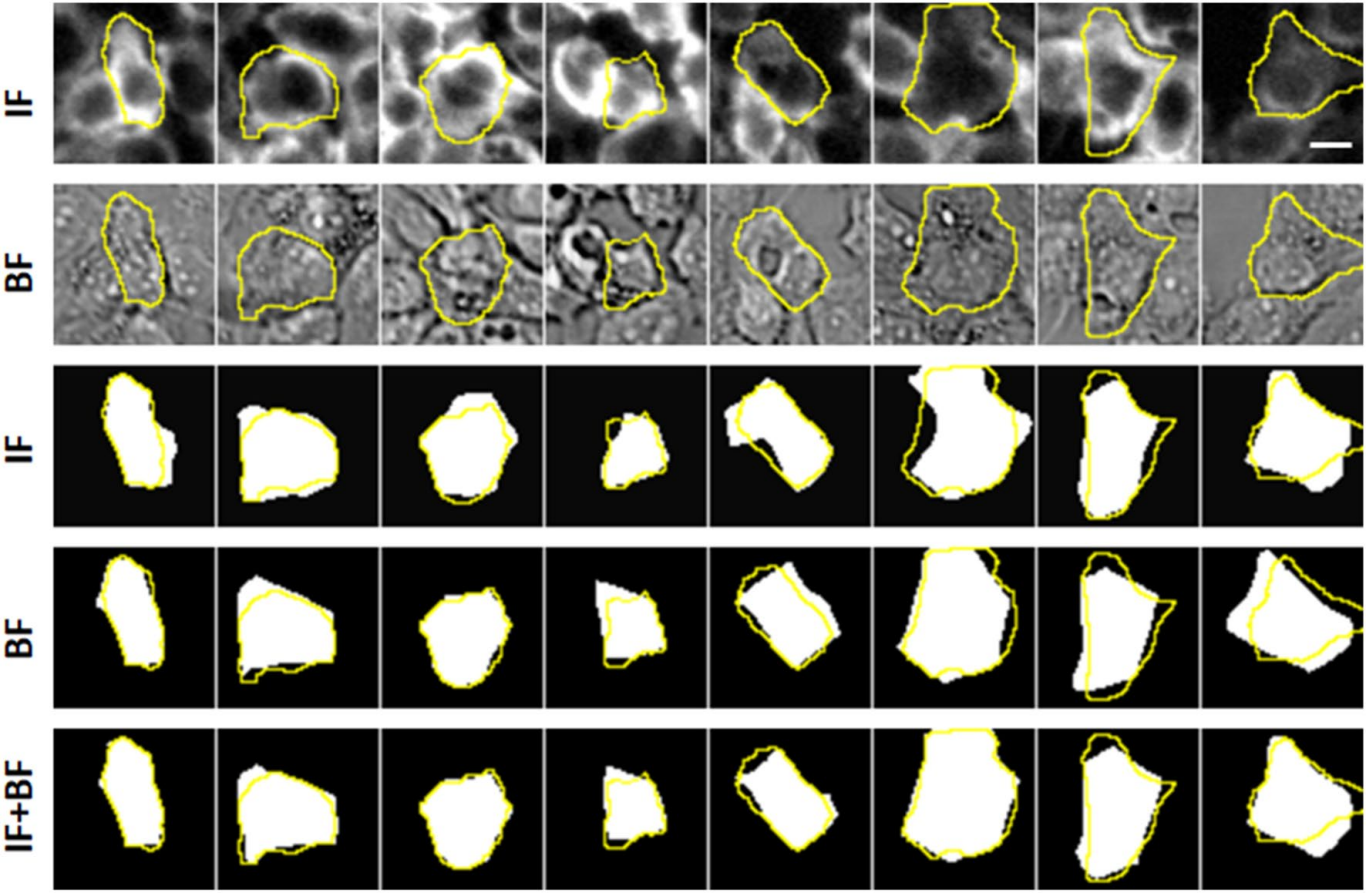
Comparisons between machine and manual segmentation results. Image patches of the Anti-pY immunofluorescence (IF, first row) and bright-field (BF, second row) microscopy data from A431 cells were used as inputs to train the segmentation model in an unsupervised manner. The bottom three rows show the CNN segmentation output of each image patch. The models were trained with either the IF (row 3), BF (row 4) or multi-channel input of IF and BF combined (row 4). In all cases, the manual segmentations of the cells were plotted as the yellow overlays. Manual segmentation is based on a combined input of IF, BF and nucleus (DAPI) images (not shown here). The scale bar represents 20 µm.

**Figure 4:**
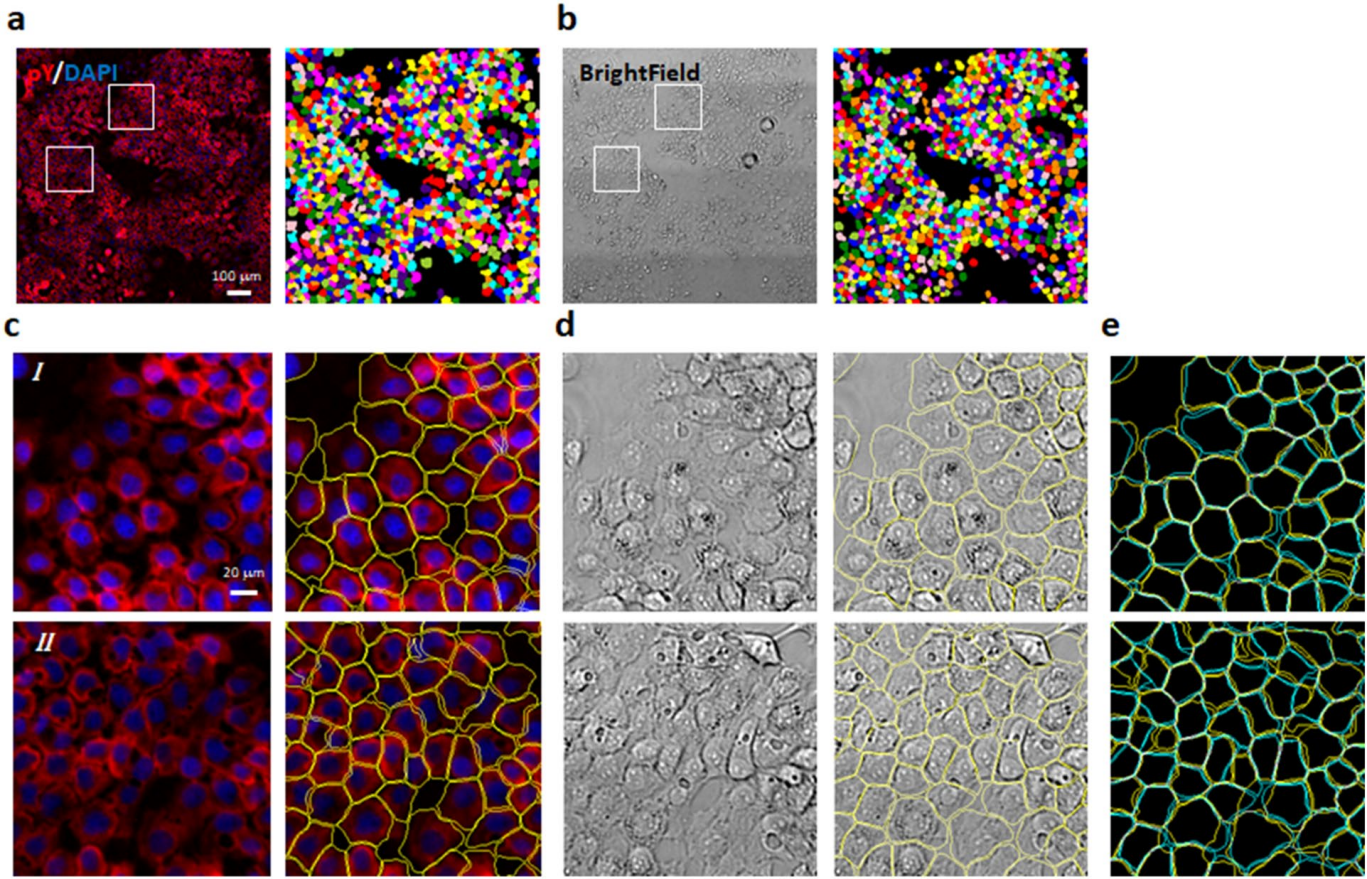
Segmentation of microscopy images by the CNN. Anti-pY immunofluorescence (**a**) and bright-field (**b**) images of A431 cells were acquired from the same sample location and the CNN segmentation results are shown as color maps (**a**,**b**, right panels). (**c**,**d**) Zoomed-in view of the segmentation results from the two sub-regions denoted by the white boxes in (**a**,**b)**. Here only the outline of the segmentations were plotted as image overlays (**c**,**d**, right columns). (**e**) Comparisons of the segmentation results based on the immunofluorescence images (yellow) versus the bright-field images (cyan). Data depict the same sub-regions as shown in **c** & **d**. All CNN segmentation models were trained with either the immunofluorescence image or the bright-field image only. DAPI images (shown in blue in **a**,**c**) were used for determining marker locations, but not used in training the CNN model.

The training process is stable and reasonably efficient. The loss function value (Fig. 2) decreases in a generally monotonic fashion until converging to a stable minimal value. The overall process took ~ 10 min on a single computational node with a P100 GPU (graphic processing unit), depending on the input. In addition, we quantified segmentation accuracies by computing mean intersection over union (mIOU) between machine segmentations and manual segmentations (Fig. 3). We found that the segmentation accuracy increases monotonically over the training process and reaches a stable maximum. Therefore, the training process did not exhibit overfitting defects, supporting the validity of the loss function design for the segmentation purpose. After the loss function value stabilized, we then integrated the single-patch outputs from the CNN to generate the segmentation map for the whole field-of-view (Fig. 4) for both immunofluorescence input and bright field input. A quick visual examination indicated that the algorithm yielded qualitatively believable segmentations in both cases.

To evaluate the segmentation quality, we performed three different quantitations of the segmentation results. First we quantified pixel level segmentation accuracy, by computing mIOUs between algorithm outputs and manual segmentations (Table 1). As expected, the modal accuracy was higher when the model had taken multi-channel data as input during training. The highest mIOU accuracy (0.825) was achieved when the model was trained with all available data (immuno-fluorescence, bright-field, and nucleus images); conversely training the models with only the bright-field images resulted in the lowest accuracy (0.701). Training with immuno-fluorescence images produced better models than training with bright-field images, which is consistent with the impression that the immuno-fluorescence data offers clearer visual cues of cell boundaries. Including the nucleus images in the model training, somewhat surprisingly, improved the model accuracy slightly. Furthermore, inclusion of the nucleus images increased the convergence speed of the training significantly (Fig. 2). This is probably because the model could (correctly) infer that a segmentation boundary should traverse through the gap between two nuclei. Thus the exact information provided by the nucleus images allows quicker learning of the semantic features from the cell images. Finally, for a baseline analysis, we performed cell segmentation on the immunofluorescence data using CellProfiler, a well-known cell segmentation software (Table 1). CellProfiler offers several predefined algorithms for unsupervised segmentation, the best of which resulted in an average mIOU value of 0.69, worse than the CNN model trained on the same fluorescence data. CellProfiler was not designed to perform segmentation on bright-field images, therefore no comparison was made for the segmentations of bright-field image data.

**Table 1:**
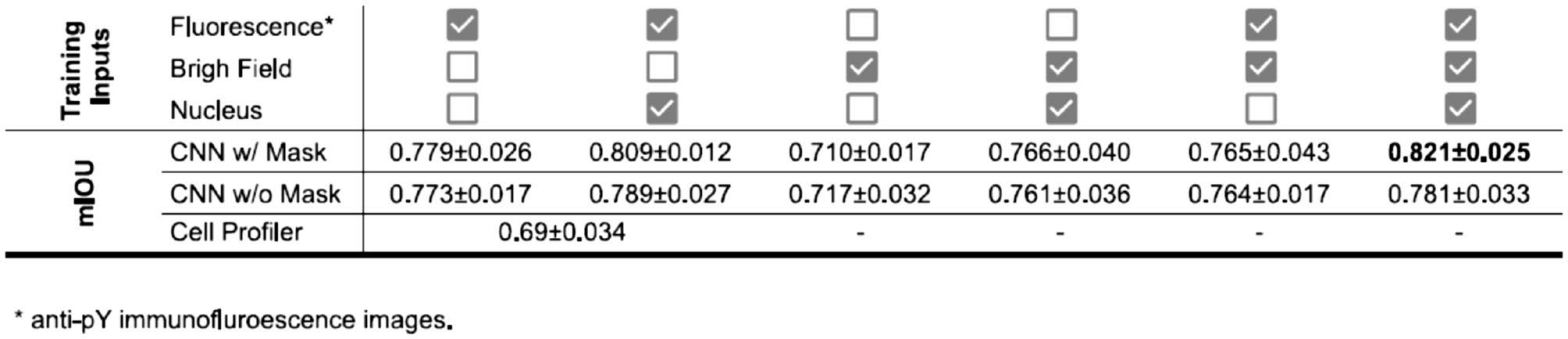
Unsupervised CNN segmentation accuracy.

Next we evaluated the consistency of segmentation results on images of differential modalities. One potential advantage of our algorithm is that it does not assume a priori specific characteristics of the data, and instead try to “learn” the semantic features from the images themselves. As a result the same algorithm could perform segmentation on either the fluorescence images or the bright-field images (Fig. 4) without the need to change model structure or hyperparameters. If the segmentation results were accurate, then the results should be similar, if not identical, on images representing the same sample, which we found to be the case (Fig. 4e). Quantitatively, we found that the mIOU between two segmentation results, one on the anti-pY immunofluorescence images and the other on bright-field images, was 0.74 ± 0.017. The mIOU value increased to 0.809 ± 0.051, if the nucleus images were also included during the model training. Therefore the segmentation outputs on differential image modalities indeed yielded results similar to one other, further supporting the notion that the self-supervised training process allows the CNN to learn correct cellular features.

Additionally, we tested whether models trained with one image input can perform segmentation on a different image that it had not seen yet (Figs. S4 and S5). If the unsupervised training procedure allows the model to learn semantic features, then we should reasonably expect the trained model to also be able to operate on a different image that is acquired in a similar manner. Indeed we found that a pre-trained model can segment an unseen image producing a segmentation similar to that of a “proper” model, which had previously seen the image (Figs. S4 and S5), with mIOU = 0.752 for anti-pY immunofluorescence images and mIOU = 0.772 for bright-field images.

**Figure 5:**
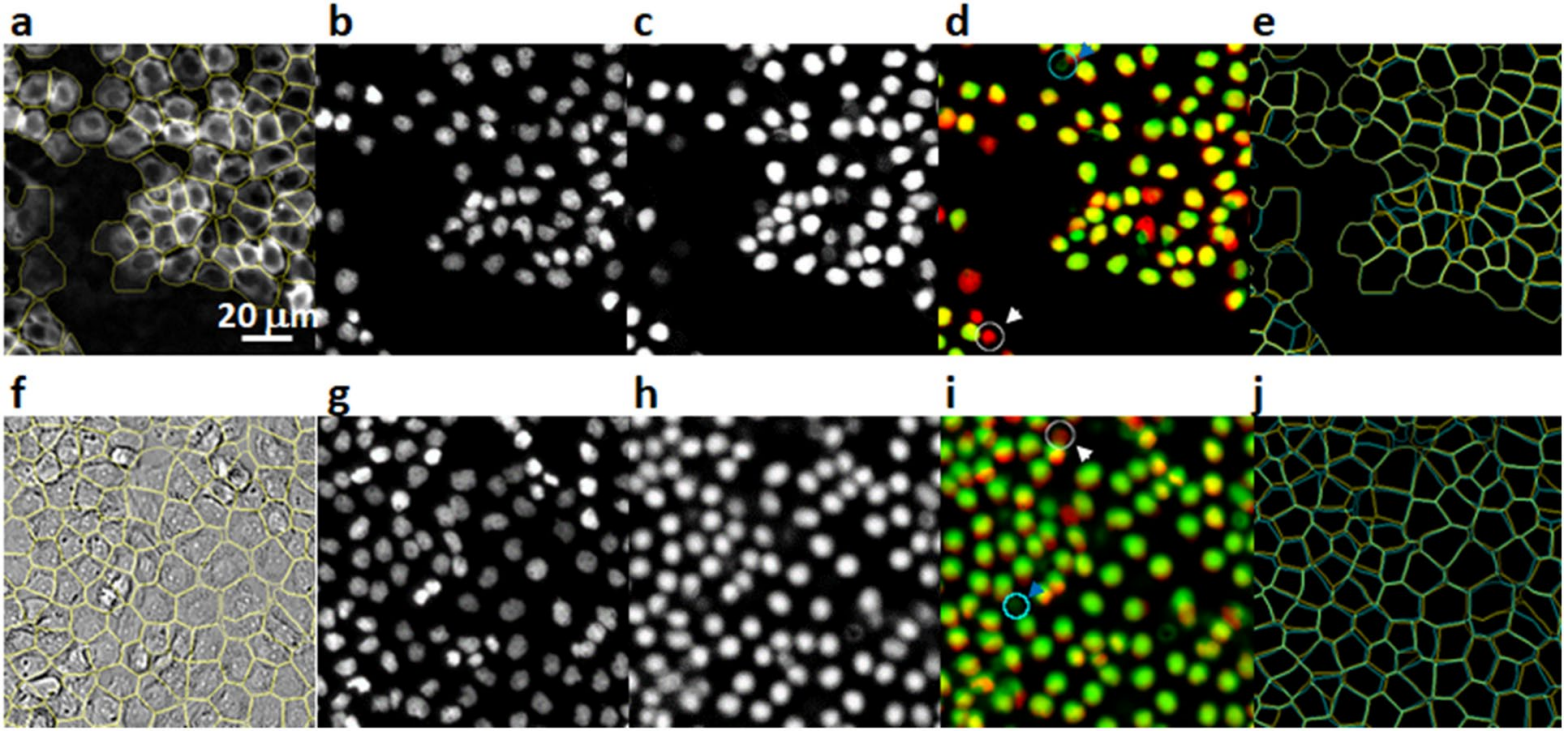
Segmentation based on synthetic marker locations. Results for the anti-pY immunofluorescence data are shown in panel (**a**–**e)**, and results for the bright-field image are shown in (**f**–**j)**. The input images (**a**,**f**) were shown together with the segmentation results (yellow overlay). Experimentally acquired nucleus (DAPI) images were shown in (**b,g)** for comparison, although they were not used for the computation of the segmentation. Instead, synthetic nucleus images (**c**,**h**) were computed directly from the input images (**a**,**f**) using a pre-trained CNN. Errors in the synthetic images can be easier discerned in the composite overlay images (**d**,**i**) of the experimental (red) and synthetic (green) nucleus images. Both false negative (white arrow) and false positive (blue arrow) errors were found, but are of relatively low occurrences. Finally, the segmentation results based on synthetic markers were compared with the “proper” segmentation, for which the experimental DAPI images were used for marker locations. The comparisons were shown in (**e**,**j)**, where segmentations based on experimental markers (yellow) were drawn in overlay on segmentations based on synthetic markers (cyan).

Next, we explore the possibility of generating marker positions directly from the cellular images themselves (i.e., not requiring a nucleus image). While the existence of the nucleus image provides a convenient means to generate the marker locations, the requirement exerts extra experimental overhead. We hypothesized that marker locations can also be computationally obtained, if we produce a CNN model that could produce a synthetic “nucleus” image from a normal cellular image, e.g., a bright-field image of cells. We tested this idea by first training a UNet to perform a mapping, either from the anti-pY immunofluorescence images or from the bright-field images, to their respective nucleus images. It is important to note that our goal is not to produce a realistic nucleus image, but to compute the mark locations. Thus the model can be of relatively low resolution. Indeed, we found that a useful model can be obtained with very little training data: i.e., two images of 1750 × 1750 pixels in our test. Figure 5 shows examples of synthetic nucleus images by applying pretrained models to new images the models had not seen. While these synthetic images were far from indistinguishable from real nucleus images, they reproduced the nucleus positions with enough accuracy to allow computing marker locations via a simple blob detection algorithm. The marker locations were then combined with the original cellular images to train a segmentation model using the unsupervised procedure outlined earlier.

To evaluate the accuracy of the segmentation based on this synthetic marker procedure, we separately quantify two sources of errors. Firstly, the model generated synthetic markers did not perfectly match each individual nucleus in the sample, which resulted in either missing markers (false negative) or extra markers (false positive) at wrong locations (Fig. 5). We compared the synthetic marker locations with experimental images of the nuclei and found that, for markers generated from immunofluorescence images, the average false negative rate is 1.8 ± 0.3% and the average false positive rate is 2.2 ± 0.3%, and for markers generated from bright-field images, the average false negative rate is 2.1 ± 0.2% and the average false positive rate os 2.4 ± 0.3%. These two types of errors correspond to missing cells and over-segmentation of cells, respectively, in the final segmentation results. Furthermore, the segmentation model had additional segmentation error at pixel level, which we evaluated by comparing the single cell segmentation results with manual segmentations on selected cells. We found the mIOU value to be 0.814 ± 0.025 for segmenting anti-pY immunofluorescence images and 0.755 ± 0.033 for segmenting bright-field images. Interestingly, the segmentation errors produced here are not significantly different from the models trained with “correct” marker locations (i.e., using experimental nucleus images, see Table 1), indicating that the small amount of false-positive and false-negative errors in synthetic markers had little impact on the training of the segmentation models.

While the core component of our segmentation model is CNN-based, the overall pipeline still relies on traditional algorithms, such as Graph Cut, in order to obtain cell area masks. Therefore, there is a concern that inaccuracies in the mask input would lead to significant deteriorations of the model output. To evaluate this, we performed additional experiments in where we set the β value (Eq. 1) to zero, thereby completely eliminated any impacts of the mask data to the model output. We found that (Table 1) even without the mask input, the model can still be trained with reasonable accuracy. In fact, the mIOU measures obtained without the mask input are no more than a few percentage points lower that the full model. However, we note that this seemingly excellent results were somewhat misleading. Indeed, visual examination showed that while omitting the mask had relatively low impact on the segmentations of cells located at the interior part cell clumps, accuracy at the outer edge of the cell clumps are significantly worse (Fig. S6). This indicated that the model is very efficient at learning the appearance of cell–cell boundary. However the knowledge of cell–cell boundary does not directly translate to the recognitions of the cell-background boundaries, because the latter are typically consisted of various flattened cytoskeletal structures, such as lamellipodia, which, without extra supervision, the model has no knowledge about and therefore cannot recognize. On the other hand, only a very small portion of our cells are located at the edge of the cell clumps. Therefore the inaccuracies at these cells had a relatively low impact on the cell-averaged metrics such as mIOUs.

## Discussion

Cellular imaging based high content screening has become more widely used in biological and medical researches^27,28^. The ability to perform single cell segmentation accurately and in a cost-effective manner is of great importance both to the commercial interests and to the basic sciences of studying single cells. Currently, accurate segmentation often requires a tailored algorithm specific to a fixed imaging modality, labeling procedure and specific imaging settings. Deviation from an optimized protocol often resulted in lowered performances. In general, these requirements significantly limit the flexibility in experimental designs. Machine learning based methods, on the other hand, promise to be more flexible because they are able to learn correct semantic features directly from data. Indeed, we show here that a CNN based computation method can perform cell segmentation on inputs of vastly different signal characteristics. In comparison to conventional algorithm-based segmentation tools, our method has the advantage of having a higher accuracy and being applicable to various input signal modality. We showed that both fluorescence and bright-field microscopy images can be analyzed with this deep learning model using the same set of the hyperparameter values. More importantly, unlike previous CNN based segmentation tools, our method does not require any human labeled “ground-truth” data for training, and instead rely on an unsupervised training procedure, alleviating a significant barrier in applying deep learning strategy to cell segmentation.

The key insight from our work is that a properly designed artificial intelligence model can learn features from unlabeled cell image data by processing and comparing multiple small image patches. The general concept of patch learning was recently introduced by several highly influential studies^29,30^ of unsupervised machine learning. Most current works in this area, however, are focusing on the spatial context between non-overlapping patches as a source of free information for training the models. The training generally requires a large amount of input data. On the other hand, cellular images produced in biology have significantly higher feature density (i.e., they contain nothing but cells) than most natural images. We show that the unique characteristics of cell images can be leveraged to reduce the training data required, to a single input image in our case. Tailored to this task, our training method used overlapping image patches and relied on the consistency between patches for training, resulting in a unique algorithm for this task.

The accuracy of our method is the highest if the user also acquires nucleus images from the sample, which allows computer generation of “markers” that signify the location of individual cells. However, the experimental data is optional, as we show “synthetic” nucleus images can be generated using a machine learning approach. The latter strategy does require pre-training of a CNN, which in turn requires additional training data in the form of cellular image/nucleus image pairs. We note that nominally this is a type of supervised learning, but the training process does not require manually labeling of data, and uses experimentally acquired images as the “ground truth” instead. The overhead involved in the training process is generally low—in our case, we showed that training with two pairs of images were sufficient to produce results accurate enough for marker computation. However, the training is specific to the exact signal of interest. Switching the cellular image to a different label would need retraining of the network. In the end, users of the algorithm will decide which strategy (experimental vs synthetic nucleus markers) is more compatible with the specific research goal at hand.

## Method

### Cell culture and microscopy

All experiments were performed using the human squamous-cell carcinoma line A431 (ATCC CRL1555). Cells were plated onto plasma cleaned cover glass and grown to ~ 70% confluence in standard growth media. Cells were quickly washed once with PBS and fixed with 4% paraformaldehyde for 20 min. Cells were then washed with PBS three times for 5 min each, permeabilized in 0.1% Triton-X PBS for 10 min and washed with PBS three times for 10 min each. Dishes were then blocked in 2% BSA PBS for 60 min at 4 °C, incubated with alexa647-labeled anti-pY (1:500 in blocking buffer) for 2 h while rocking at 4 °C, washed three times for 5 min in PBS, and imaged in PBS in presence of DAPI. Epi-fluorescence and bright field images were acquired on an inverted fluorescence microscope (Olympus IX73) with a 20 × objective. An area of 1.4 × 1.4 mm was imaged with a grid pattern for each sample, resulting in stitched images of 1750 × 1750 pixels. The final images were stitched using the stitching plugin available in the Fiji software. Datasets were collected from three sample replicates.

### Binary mask

A binary mask was used during training to ensure that the segmentation of individual cells will not exceed the area covered by the cells. For fluorescence images, the binary mask is generated using the standard Graph Cut^31^ algorithm. The algorithm relies primarily on the gray value differences to separate the foreground from the background, but compensate for the over-segmentation by introducing a penalty for foreground/background boundaries. Mathematically, the algorithm solves the Markov Random Field energy minimization problem on an image with normalized intensity ${x}_{i}$:

2 $$E(x)=\sum_{i}S({y}_{i}) +\sum_{\left(i,j\right)\in C}\kappa ({y}_{i }-{y}_{j})$$

where $y\in \{\mathrm{0,1}{\}}^{N}$ is segmentation labels, $C$ is the set of all adjacent pixels pairs and $S({y}_{i})$ is the unitary energy cost, which we take to be $-log({\alpha x}_{i})$ for foreground pixels (${y}_{i}=1$), and $-log[(1-{\alpha )(1-x}_{i})]$ for background pixels (${y}_{i}=0$). The algorithm takes two parameters: $\alpha$ is a prior probability for foreground pixels, which we estimate based on the percent area covered by the cells; and $\kappa$ is the constant cut penalty, for which we use a fairy large value^10^ to prevent over-segmentation.

For bright-field images, the gray values are not useful for distinguishing the foreground from the background. Therefore, we first train a two-class Random Forest model using the “Weka Segmentation”^32^ plugin in ImageJ. For both the foreground (cell region) and the background, we draw 5–6 scribbles on the input image to define the pixels used for training. We used Gaussian filter, variance and Laplacian values as feature inputs. We then performed a graph cut on the model’s probability output and used the results as the mask.

### Segmentation model

We first obtain a list of marker positions according to the centroid locations of nuclei, using CellProfiler (for experimental nucleus images) or using a blob detection algorithm (for synthetic nucleus images). Image patches were extracted from the inputs, which were either anti-pY fluorescence images or bright-field images of cells. A modified UNet (Fig. S1), used as the engine for cell segmentation, was trained to minimize the custom loss function (Eq. 1). The value for the hyperparameter $\lambda$ is chosen based on consideration of the specific case of ${y}^{j}=0.5$, in which the second term simplify to:

3 $$-\lambda \sum_{{d}_{0},{d}_{1}}\sum_{i\ne j}{y}_{{d}_{0},{d}_{1}}^{i}\mathrm{log}\,0.5\approx \frac{\lambda }{3.32}\sum_{{d}_{0},{d}_{1}}\sum_{i\ne j}{y}_{{d}_{0},{d}_{1}}^{i}$$

In other words, $\lambda =3.32$ can be considered as a “balanced” weight, because in such a case, the second term cancels out the first term when the segmentation probability from another patch is a coin-toss. Additionally, we chose a high value of $\beta$=15, to ensure a stiff penalty preventing segmentation of “background” pixels outside the masked area.

The CNN model was trained using the adaptive momentum estimation (ADAM) optimization algorithm with a constant learning rate of 0.005. Because the training can be considered as unsupervised, all image data were combined as the training set without splitting. Typically, processing all available image patches in one batch would exceed the memory capacity of a GPU, even if the patches are from just one input image. Therefore, we limit each computational batch to patches within a 640 × 640 area of the input image and process the whole image in steps. Data augmentation was implemented by randomly flipping and/or transposing the input patches. A corresponding reverse operation was performed on the network output before the computation of the loss function. The network was trained until the loss function reaches a stable minimum. To integrate individual segmentation results from patches to yield the segmentation results for the whole field-of-view, each pixel was assigned to the cell with the highest probability output at that location. Pixels showing segmentation probabilities less than 0.5 from all patches were assigned as the background.

### Synthetic nucleus image

The same UNet architecture was used as the base CNN model for producing synthetic nucleus images. We use a three-fold cross-validation scheme for evaluating the training results. In other words, the UNet models were trained with either two immunofluorescence (anti-pY) images or two bright field images, paired with corresponding nuclei images, which were thresholded and converted to binary images. We use the remaining one image pair for evaluation. The training used the cross-entropy loss function and the ADAM optimizer with a learning rate of 0.005. We stopped the training when the loss function reached the value of 0.15. The pre-trained model was then applied to the unseen images to produce the synthetic nucleus images. To evaluate the accuracy of the synthetic markers, we used a simple blob detection algorithm^33^, based on the difference of Gaussians operator^34^, to detect the location of each nucleus. The algorithm was applied to both the experimental and synthetic nuclei images and we paired a synthetic marker with a true marker if the two are within 15 µm distance. Both false positive rates and false negative rates were computed afterwards.

## Supplementary Information

Supplementary Figures.

## Data Availability

Current implementation of the segmentation algorithm is available as a github repository. https://github.com/jiyuuchc/cellcutter.
