# Supplementary material for: Training a deep learning model for single-cell segmentation without manual annotation
Source: Sci Rep. 2021 Dec 14;11:23995. doi: 10.1038/s41598-021-03299-4 (PMC8671438; doi:10.1038/s41598-021-03299-4)
Supplement: Supplementary file 1 — Supplementary Figures. [file 41598_2021_3299_MOESM1_ESM.docx]

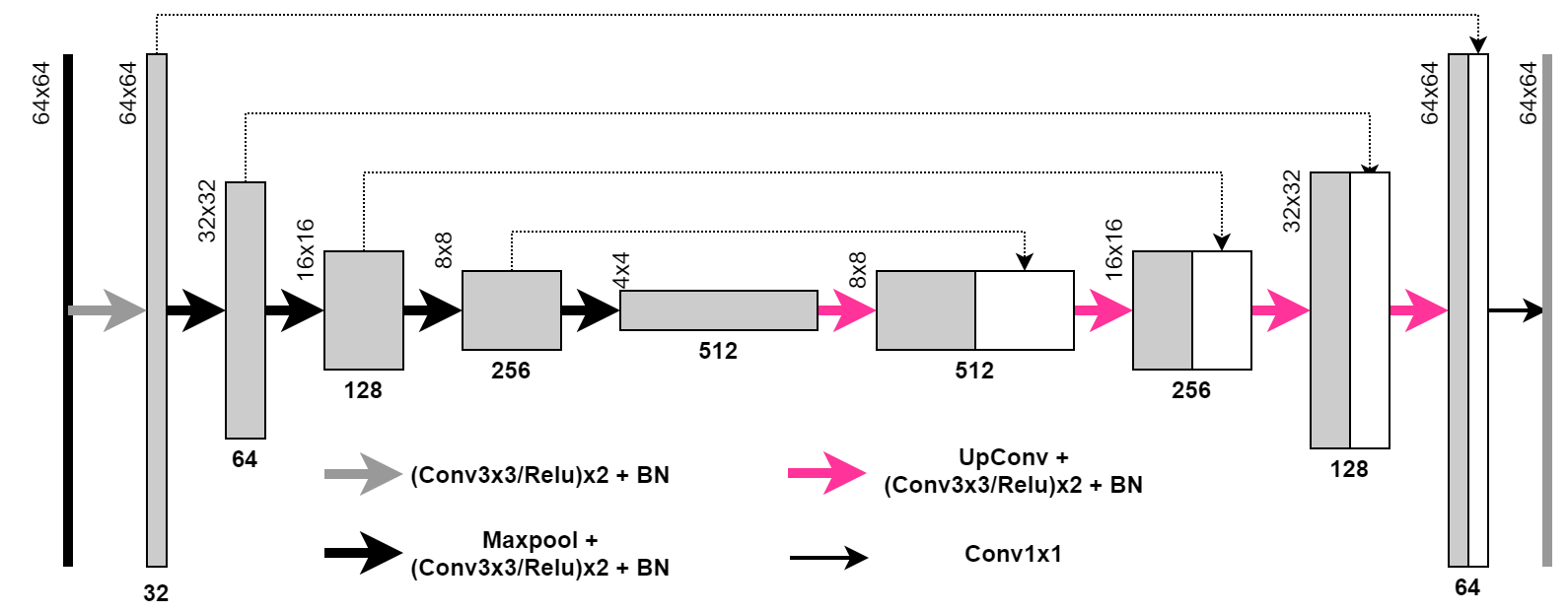


**Figure S1.** Architecture of the CNN used f or segmentation. The encoder portion of the network extracts image features by multiple stages of convolution and max pooling. The decoder portion of the network constructs the segmentation by incremental upscaling of the feature map (to 2x larger dimension) followed by convolution. Feature map obtained in the decoding step is concatenated by intermediate results at the decoding steps in order to supplement feature information at incrementally higher resolution. Relu activation is used throughout the encoding and decoding steps, except in the final output layer, in which a sigmoid activation is used. The same network is used for producing synthetic nucleus images, in which case the input tensor dimension is much larger (870x870) to match the size of training data.

**Figure S2**. Principle of the segmentation CNN training. We use a marker-guided training paradigm. Instead of training the CNN using the whole image, small patches of cell images are batched and used to train the CNN. The figure illustrate potential results from the CNN if two image batches were used as the input. Both the top and bottom results were not optimal based on the loss function Eq 1. The bottom solution has conflicting output from two patches and incur penalty due to the second terms of the loss function. The top solution has no conflict but has suboptimal segmented area, and thus incur penalty via the first term of the loss function.

**Figure S3.** Examples of the binary masks produced in the image preprocessing step. (a) and (c) show the input fluorescence (a) and bright field (c) microscopy images. (b) and (d) show the corresponding binary masks. The mask for the fluorescence image were computed via Graph Cut algorithm, using image intensity as the relative weights for foreground/background assignment. The mask for the bright field image was also computer from Graph Cut, but instead of using image intensity directly, weights were computed using a Random Forest model. The model was trained interactively using the WEKA plugin in ImageJ. See main text for more details.

**Figure S4**. Comparison of segmentation results on both seen and unseen fluorescence images. Two segmentation models were separately trained by two immunofluorescence images of the same modality (left) and the resulting models were used to operate on both images (one seen and one unseen) to obtain the segmentation (right). The results demonstrated that the models trained this way were able to apply l earnings to unseen data of similar features

**Figure S5.** Comparison of segmentation results on both seen and unseen bright-field images.

This is the same analysis as in Fig S4 except on bright-field images.

**Figure S6.** Effects of omitting mask input. Segmentation of bright-field (a) and fluorescence (b) cell images without mask input, by setting the hyper-parameter β to zero. The results show model inaccuracies for cells at the edge of the cell colonies. However, segmentations of cells in the interior of the colonies are largely unaffected.
